# Vitamin D supplementation for patients with alopecia areata: A protocol for systematic review and meta-analysis

**DOI:** 10.1097/MD.0000000000031089

**Published:** 2022-10-21

**Authors:** Baohua Zhu, Lan Zhang, Jun Wang, Guiyuan Tan

**Affiliations:** a Hospital of Chengdu University of Traditional Chinese Medicine, Chengdu, 610075, Sichuan Province, China; b Nanjing University of Traditional Chinese Medicine, Nanjing, Jiangsu Province, China.

**Keywords:** alopecia areata, meta-analysis, protocol, systematic review, vitamin D

## Abstract

Several studies have reported an association between low Vitamin D (VD) levels and Alopecia areata (AA), and suggested VD preparations as a potential therapy. VD has immunomodulatory function closely associated with a variety of skin diseases, but there is no conclusive evidence to support VD supplementation for patients with AA. Therefore, we will conduct a meta-analysis to collect and evaluate the efficacy and safety of VD supplementation in the treatment of AA.

**Methods:**

We will search the following databases according to the developed strategy: Cochrane Library, PubMed, Web of Science, EMBASE, Scopus, Google scholar for Randomized controlled trials related to VD supplementation for AA. The retrieval time is from the establishment of each database to July 2022. Two reviewers will independently complete the literature search and screening, risk of bias assessment and data extraction. Severity of Alopecia Tool, Alopecia Density and Extent, Lesional area and senstivity (score) will be the primary results. RevMan V.5.3 will be used for data analysis and synthesis. For dichotomous outcomes and the continuous outcomes, we will calculate risk ratio with 95% Confidence intervals and mean differences or standardized mean differences with 95% Confidence intervals respectively. The reporting bias will be investigated using funnel plots, and the asymmetry of the funnel plots will be explained using the Harbord modified test or the Egger’s regression test.

**Results:**

The results of the study expect to provide a high-quality, evidence-based recommendation on VD supplementation in the treatment of AA for clinicians.

**Conclusions:**

The study will provide scientific and useful evidence for better use of VD supplementation in treating AA.

## 1. Introduction

Alopecia areata (AA) is a type of T cell-mediated autoimmune disease involving hair follicles (HF), which is manifested as round or oval non-cicatricial alopecia on the head, but also can occur anywhere in the body. The global morbidity of AA ranges from 0.1% to 0.2%, affecting the population of all ages without significant difference between genders.^[1]^

AA is mainly treated topically, including intralesional hormone injections, contact immunotherapy, JAK inhibitors, minoxidil, the excimer laser/light and so on.^[2]^ Intralesional or local corticosteroid is the most effective treatment for AA, Intralesional or topical corticosteroids are the most effective treatments for AA, but they often cause adverse effects such as folliculitis, skin atrophy, and osteoporosis.^[3]^ Moreover, the adverse events of other treatment methods have also been reported. For example, sensitization after diphenyl prone immunotherapy may lead to severe head and face edema.^[4]^ JAK inhibitors are prone to cause complications such as upper respiratory tract infection, elevating transaminase, and headache.^[5]^ Long-term phototherapy can cause pigmentation, photoaging, and even canceration.^[6]^ Pain is common occurred in patients receiving platelet rich plasma.^[5]^ Each treatment has its advantages and disadvantages and consequently providing a more safe, effective, and economic treatment for AA patients is a hard issue in current research.

Serum 25-(OH)D is the best biological indicator of vitamin D (VD). Accumulating evidence has shown that the serum 25-(OH) D level of AA patients is decreased, which is negatively correlated with the severity, course of the disease, and the number of plaques.^[7–9]^ VD constitutes 1 of the most important sterol derivatives in the human body, which mainly regulates the metabolism of calcium and phosphorus. It is closely related to a variety of common skin diseases, such as atopic dermatitis, acne, psoriasis, vitiligo, androgen alopecia, and AA.^[10]^ A study suggested that lack of sunshine caused by social phobia and fewer outdoor activities may lead to the decrease of VD in severe AA patients.^[11]^

1,25(OH)_2_D_3_, an active form of VD, exerts different effects on different subpopulations of T lymphocytes, which can inhibit the proliferation and differentiation of Th1 cytokines (IFN-*γ*, TNF-*α*, IL-2), induce the expression of Th2 cytokines (IL-4, IL-5, IL-10), as well as promote the polarization of Th1 to Th2 immune response.^[12]^ Also, it inhibits the activation and maturation of B cells and dendritic cells and facilitates the differentiation of regulatory T cells (Tregs),^[12]^ thereby inducing immune tolerance and reducing the risk of autoimmune diseases. All these are conducive to alleviating inflammation and restoring HF privilege.

AA is often complicated with depression, anxiety, social phobia, and obsessive-compulsive disorder, and these mental diseases may have occurred months or even years before the onset of AA.^[13]^ VD is also a neurosteroid hormone. It has been reported that VD activates the transcription of tryptophan hydroxylase 2 to promote the release of 5-hydroxytryptamine, similar to antidepressants such as fluoxetine hydrochloride.^[14]^ Besides, VD enhances the gene expression of tyrosine hydroxylase, which is the rate-limiting step of catecholamine synthesis.^[15]^The catalytic synthesis of these neurotransmitters contributes to correcting mood disorders.

Oxidative stress (OS) is caused by excessive production of reactive oxygen species and insufficient antioxidants. A systematic review and meta-analysis demonstrated that the levels of serum malondialdehyde, nitric oxide and total oxidant were increased, while superoxide dismutase, catalase and glutathione peroxidase were reduced in patients with AA.^[16]^ OS induces the expression of MICA (MHC class I-related chain A) in HF cells, which leads to the collapse of immune privilege and T cell attack.^[16]^ VD supplementation is indirectly involved in the synthesis of antioxidants and prevent OS by regulating the expression of Klotho and Nrf2.^[16]^

Having the features as low cost and ease of administration, VD can be administered to the patients with AA. In general, VD supplementation is safe and VD intoxication is very rare. Furthermore, the prospective studies or systematic reviews on the improvement of immune-related skin diseases such as atopic dermatitis, psoriasis and vitiligo by VD supplementation have been carried out continuously.^[17–19]^

Therefore, it is necessary to design this systematic evaluation and meta-analysis based on the latest evidence to determine the effectiveness and safety of VD supplementation in the treatment of AA. Providing more scientific and cutting-edge evidence for AA treatment option will be our ultimate aim.

## 2. Methods

### 2.1. Protocol and registration

According to the standard of Preferred Reporting Items for Systematic Review and Meta-Analysis Protocols,^[20]^ we registered it in PROSPERO (CRD42022349766).

### 2.2. Inclusion criteria

#### 2.2.1. Types of studies.

Randomized controlled trials (RCTs) that examine the efficacy and safety of VD supplementation on AA patients will be included. Considering the time required for hair growth, the study period is generally not less than 3 months.^[21]^ In addition, there should be adequate data of serum 25 (OH) D level. Studies inconsistent with the research content, no RCTs, lack of patients’ outcomes or measurement methods of the trial, study without the full text or valid data, literature reviews, conference proceedings, animal experiments, duplicate reports will be excluded.

#### 2.2.2. Types of participants.

We will include patients diagnosed with AA, either by direct visualization, trichoscopy or skin biopsy. Other criteria need to be met as follows:

1. Laboratory parameter: at least serum 25(OH)D concentrations < 50 ng/mL (125 nmol/L),^[22]^ qualified liver and kidney function.
2. There is no evidence of obvious spontaneous regrowth of terminal hair.
3. Detailed medical history and physical examination will be performed to exclude cicatricial alopecia, such as pseudo alopecia areata, localized scleroderma, discoid lupus erythematosus, lichen planus, tinea capitis, alopecia folliculitis or trichotillariasis, and telogen alopecia.

#### 2.2.3. Type of interventions.

##### 2.2.3.1. Experimental interventions.

The experiment of VD or an active form of VD supplementation alone or in combination with another active treatment (drug or non-drug) will be included. No dosage, usage, frequency and duration restrictions will be applied.

##### 2.2.3.2. Comparator interventions.

The control group included nonintervention, placebo, other treatments, or the same combination therapy as the experimental group but did not contain VD or VD analogues.

#### 2.2.4. Types of outcome measures.

##### 2.2.4.1. Primary outcomes.

Methods for assessing severity of hair loss in AA are the primary outcomes. Severity of Alopecia Tool score,^[23]^ which categorizes the scalp terminal hair loss into 6 grades (S0-S5). Others like Alopecia Density and Extent score, Lesional area and senstivity score will be evaluation indexs too.^[23]^

##### 2.2.4.2. Secondary outcomes.

The secondary outcomes will mainly include the following aspects: serum or tissue VD level, Alopecia Areata Progression Index,^[24]^ various quality of life assessment forms^[23]^(Skindex-16, Dermatology Life Quality Index, Alopecia Areata Symptom Impact Scale etc).

##### 2.2.4.3. Adverse events

The number and causes of adverse events will be recorded.

### 2.3. Search strategy

The published studies will be identified through systematic searches of the following electronic databases: Cochrane Library, PubMed, Web of Science, EMBASE, Scopus, Google scholar. The retrieval time limit is from all databases establishment to July, 2022. The search will be performed by combining subject terms with free words. There are no restrictions on language or publication date. The search strategy for Cochrane Library was showed in Table 1. We will also complete the rest of the search based on specific requirements of other databases. In addition, other sources (ClinicalTrials.gov, International Clinical Trials Registry Platform) will be searched to ensure the comprehensiveness of the study.

**Table 1:** The search strategy used in Cochrane Library.

	Search strategy for The Cochrane Library
#1	MeSH descriptor: [Vitamin D] explode all trees
#2	MeSH descriptor: [Ergocalciferols] explode all trees
#3	(“Calciferols”):ti,ab,kw OR (“D2, Vitamin”):ti,ab,kw OR (“Vitamin D2”):ti,ab,kw OR (“Ergocalciferol”):ti,ab,kw OR (“Vitamin D2”):ti,ab,kw
#4	#2 OR #3
#5	MeSH descriptor: [Cholecalciferol] explode all trees
#6	(“(3 beta,5Z,7E)-9,10-Secocholesta-5,7,10(19)-trien-3-ol”):ti,ab,kw OR (“Calciol”):ti,ab,kw OR (“Vitamin D 3”):ti,ab,kw OR (“Vitamin D3”):ti,ab,kw OR (“Cholecalciferols”):ti,ab,kw
#7	#5 OR #6
#8	MeSH descriptor:[Calcitriol] explode all trees
#9	(“Sitriol”):ti,ab,kw OR (“Rocaltrol”):ti,ab,kw OR (“1 alpha,25-Dihydroxyvitamin D3”):ti,ab,kw OR (“1,25-Dihydroxyvitamin D3”):ti,ab,kw
#10	(“D3, 1 alpha,25-Dihydroxyvitamin”):ti,ab,kw OR (“1,25 Dihydroxyvitamin D3”):ti,ab,kw OR (“1 alpha,25-Dihydroxycholecalciferol”):ti,ab,kw
#11	(“1 alpha,25 Dihydroxyvitamin D3”):ti,ab,kw OR (“1,25 Dihydroxycholecalciferol”):ti,ab,kw OR (“D3, 1,25-Dihydroxyvitamin”):ti,ab,kw
#12	(“1,25-Dihydroxycholecalciferol”):ti,ab,kw OR (“Bocatriol”):ti,ab,kw OR (“Renatriol”):ti,ab,kw OR (“Calcitriol KyraMed”):ti,ab,kw
#13	(“KyraMed, Calcitriol”):ti,ab,kw OR (“Osteotriol”):ti,ab,kw OR (“Calcijex”):ti,ab,kw OR (“Decostriol”):ti,ab,kw OR (“Tirocal”):ti,ab,kw
#14	(“MC 1288”):ti,ab,kw OR (“MC1288”):ti,ab,kw OR (“MC-1288”):ti,ab,kw OR (“Calcitriol-Nefro”):ti,ab,kw OR (“Calcitriol Nefro”):ti,ab,kw
#15	(“1,25-dihydroxy-20-epi-Vitamin D3”):ti,ab,kw OR (“1,25 dihydroxy 20 epi Vitamin D3”):ti,ab,kw OR (“D3, 1,25-dihydroxy-20-epi-Vitamin”):ti,ab,kw
#16	(“1 alpha, 25-dihydroxy-20-epi-Vitamin D3”):ti,ab,kw OR (“1,25(OH)2-20epi-D3”):ti,ab,kw OR (“20-epi-1alpha,25-dihydroxycholecaliferol”):ti,ab,kw
#17	(“Silkis”):ti,ab,kw OR (“Soltriol”):ti,ab,kw
#18	#8 OR #9 OR #10 #11 OR #12 OR #13 #14 OR #15 OR #16 #17
#19	(“alfacalcidol”):ti,ab,kw OR (“1α-hydroxyvitamin D”):ti,ab,kw OR (“25-hydroxyvitamin D ”):ti,ab,kw OR (“calcidiol”):ti,ab,kw
#20	#1 OR #4 OR #7 #18 OR #19
#21	MeSH descriptor: [Alopecia Areata] explode all trees
#22	(“Alopecia Circumscripta”):ti,ab,kw
#23	#21 OR #22
#24	MeSH descriptor: [Randomized controlled trial] explode all trees
#25	(“Randomized controlled trial”):ti,ab,kw OR (“Randomized”):ti,ab,kw OR (“controlled”):ti,ab,kw
#26	#24 OR #25
#27	#20 AND #23 AND #26

### 2.4. Collection and analysis of data

#### 2.4..1. Selection of studies.

**Figure 1.**
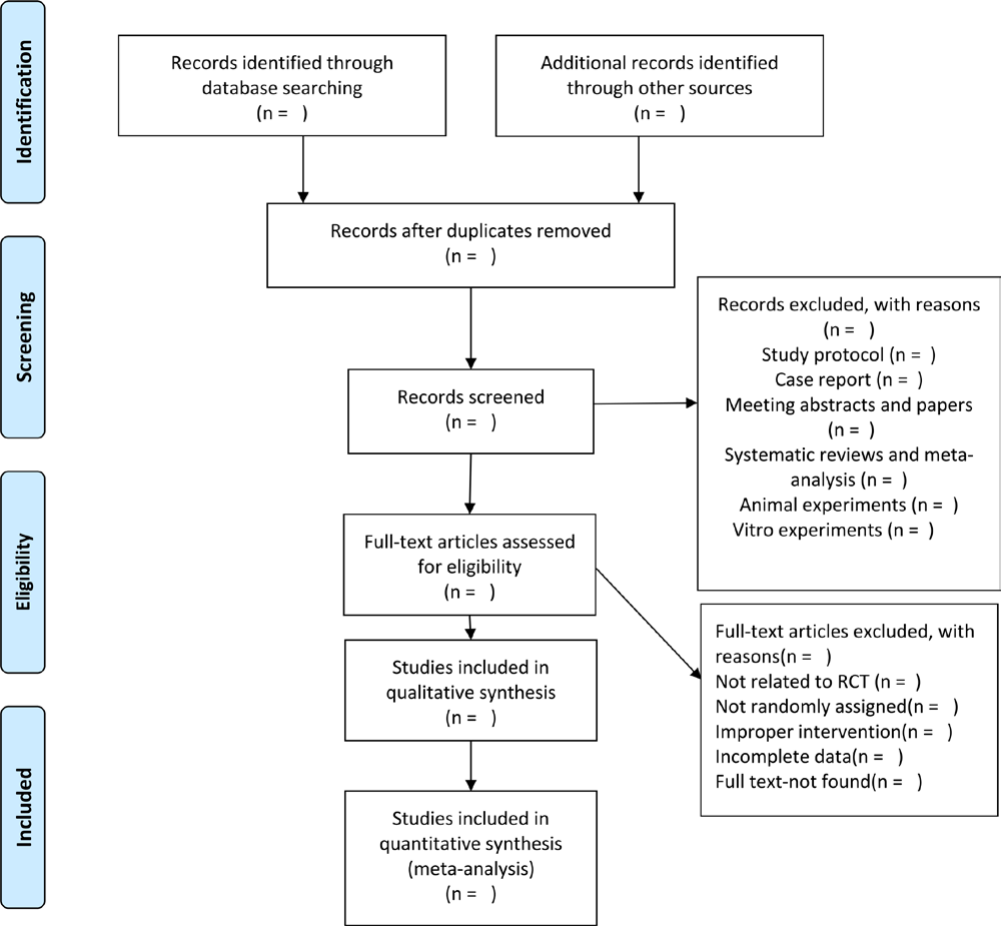
Flow diagram of the study selection process.

Note Express Soft (Beijing, China) will be used for literature management. After eliminating the duplicate literature, 2 trained reviewers will identify potentially eligible studies that meet the inclusion criteria based on the title and abstract. They will investigate all of them as full text and screen out the final included studies. In this process, 2 reviewers are required to complete the task independently. Reasons for the discarded studies will be noted in detail. Possible differences will be discussed and resolved by consulting with a third reviewer if necessary. The process and results of studies selection will be presented in a Preferred Reporting Items for Systematic Review and Meta-Analysis diagram (Fig. 1).^[25]^

#### 2.4.2. Data extraction and management.

Two reviewers will independently extract relevant data from the included studies using standardized forms and enter them into STATA 16.0. A third reviewer will check for consistency. The following information will be recorded:

1. General information: title, author, location, setting, publication date, clinical trial registration, fundings;
2. Study characteristics: design, randomization, allocation concealment, blind method, other concerns about bias, inclusion and exclusion criteria;
3. Patients characteristics: sample size, average age, gender, race, type and severity of AA;
4. Intervening measure: total number of intervention groups, form, mode of administration, dosage, frequency, duration of VD supplementation, co-intervention (if any);
5. Outcomes: primary and secondary outcomes, adverse events, valid unit of measurement.

#### 2.4.3. Assessment of risk of bias in included studies.

We will use the Cochrane “bias risk” tool^[26]^ to assess the bias risk of the quality of the included literature. Assessment items included random sequence generation, allocation concealment, blinding of participants and personnel, blinding of outcome assessment, whether the incomplete outcome data were adequately handled, evidence of selective outcome reporting, and other potential sources of bias. The RevMan V.5.3 software provided the visualized figures after bias risk assessment.

#### 2.4.4. Measures of treatment effect.

According to Cochrane Handbook for Systematic Reviews of Interventions,^[27]^ risk ratio or mean difference will be calculated and presented for statistical analysis of binary and continuous variables respectively. If different measurement methods are employed for the same result, standardized mean difference will be used to assess the intervention effect.^[27]^ A 95% Confidence interval will be provided for each effect size.^[27]^ The *P* < .05 is considered statistically significant.

#### 2.4..5. Dealing with missing data.

We will try to get in touch with the trial authors through the email or telephone provided by their manuscript to request the unclear or missing data. Imputation will be used to address missing data if the problem cannot be solved.^[28]^

#### 2.4..6. Assessment of heterogeneity.

Chi-square test and *I*^2^ statistic will be used for assessing the statistical heterogeneity.^[29]^ If *P* < .1 or *I*^2^ > 50%, it is considered that there is significant heterogeneity among the included studies. Next, we will determine the potential reasons for heterogeneity by performing sensitivity or subgroup analysis.

#### 2.4.7. Assessment of publication biases.

The reporting bias will be investigated using funnel plots visually if ≥ 10 RCTs are included in a meta-analysis.^[30]^ For dichotomous outcomes and continuous outcomes, Harbord modified test and Egger’s regression test will be used to explain the asymmetry of the funnel plots respectively (if any).^[31]^

### 2.5. Data synthesis

RevMan V.5.3 software will be used to analyze all data. In meta-analysis, Mantel-Haenszel method will be conducted to estimate the binary outcomes effect size, while Inverse-Variance method will be conducted to estimate the continuous outcomes effect size. We will use the fixed-effect model to pool data whenever there is low heterogeneity. Analysis and treatment will be carried out first whenever there is high heterogeneity (*P* < .1 or *I*^2^ > 50%). If it cannot be solved, the random-effect model will be introduced to provide a more conservative effect estimation. For the research results with large heterogeneity that cannot be quantitatively integrated, a narrative report will be made.

#### 2.5.1. Subgroup analysis and investigation of heterogeneity.

Considering that the VD level in vivo is affected by multiple factors, we will conduct subgroup analysis under the premise of sufficient data according to the basic information of patients, such as age, gender, weight, region (latitude), baseline serum 25-(OH)D level(<25 or ≥ 25 nmol/L), course of the disease, severity and type of AA, and past history of alopecia, thereby investigating the source of heterogeneity. The type of VD supplementation, mode of administration (oral or intramuscular), dosage, and frequency in the experimental group, or the measures (placebo, other drugs or methods) in the control group are also the stratification factors we consider.

#### 2.5.2. Sensitivity analysis.

Sensitivity analysis is mainly used to evaluate the robustness and reliability of meta-analysis combined results. In future updates as more data become available, we will conduct it according to the following criteria: sample size, literature quality ratings, selected statistical model, missing data.

## 3. Discussion

In addition to immune disorder, the pathogenesis of AA includes heredity, mental stress, infection, OS, and microcirculation disorder. VD supplementation can not only directly solve the problem of micronutrient deficiency, but also indirectly alleviate AA from various aspects. VD deficiency is common in AA, but there is limited evidence to determine whether VD supplementation improves AA currently. Thus, this study will provide high-quality evidence-based medicine to evaluate the possible benefit of VD supplementation for patients with AA and establish cause-and-effect relationships even.

## Author contributions

**Conceptualization:** Baohua Zhu.

**Data curation:** Jun Wang.

**Formal analysis:** Lan Zhang, Baohua Zhu.

**Methodology:** Baohua Zhu, Jun Wang.

**Project administration:** Guiyuan Tan.

**Supervision:** Guiyuan Tan.

**Validation:** Jun Wang, Lan Zhang.

**Writing – original draft:** Baohua Zhu.

**Writing – review & editing:** Lan Zhang.

## References

[1] Strazzulla LC Wang EHC Avila L. Alopecia areata: Disease characteristics, clinical evaluation, and new perspectives on pathogenesis. J Am Acad Dermatol. 2018;78:1–12. 29241771 10.1016/j.jaad.2017.04.1141

[2] Darwin E Hirt PA Fertig R. Alopecia areata: review of epidemiology, clinical features, pathogenesis, and new treatment options. Int J Trichology. 2018;10:51–60. 29769777 10.4103/ijt.ijt_99_17 PMC5939003

[3] Trüeb RM Dias MFRG. Alopecia areata: a comprehensive review of pathogenesis and management. Clin Rev Allergy Immunol. 2018;54:68–87. 28717940 10.1007/s12016-017-8620-9

[4] Lamb RC Young D Holmes S. Retrospective review of diphencyprone in the treatment of alopecia areata. Clin Exp Dermatol. 2016;41:352–8. 26620737 10.1111/ced.12776

[5] Almohanna HM Perper M Tosti A. Safety concerns when using novel medications to treat alopecia. Expert Opin Drug Saf. 2018;17:1115–28. 30318935 10.1080/14740338.2018.1533549

[6] Vaani VV Tang MM Tan LL. The utilization of phototherapy in the department of dermatology, Hospital Kuala Lumpur: a 5-year audit. Med J Malaysia. 2018;73:125–30. 29962494

[7] Liu Y Li J Liang G. Association of alopecia areata with vitamin D and calcium levels: a systematic review and meta-analysis. Dermatol Ther (Heidelb). 2020;10:967–83. 32772238 10.1007/s13555-020-00433-4 PMC7477029

[8] Daroach M Narang T Saikia UN. Correlation of vitamin D and vitamin D receptor expression in patients with alopecia areata: a clinical paradigm. Int J Dermatol. 2018;57:217–22. 29243839 10.1111/ijd.13851

[9] Unal M Gonulalan G. Serum vitamin D level is related to disease severity in pediatric alopecia areata. J Cosmet Dermatol. 2018;17:101–4. 28447433 10.1111/jocd.12352

[10] Navarro-Triviño FJ Arias-Santiago S. Vitamin D and the skin: a review for dermatologists. Vitamina D y la piel. Una revisión para dermatólogos. Actas Dermosifiliogr. 2019;110:262–72. 30857638 10.1016/j.ad.2018.08.006

[11] Yao CA. Serum vitamin D level and disease severity of alopecia areata: a meta-regression analysis. J Am Acad Dermatol. 2018;79:e49–50. 29753061 10.1016/j.jaad.2018.05.009

[12] Bishop E Ismailova A Dimeloe SK. Vitamin D and immune regulation: antibacterial, antiviral, anti-inflammatory [published online ahead of print, 2020 Aug 22]. JBMR Plus. 2020;5:e10405. 32904944 10.1002/jbm4.10405 PMC7461279

[13] Chu SY Chen YJ Tseng WC. Psychiatric comorbidities in patients with alopecia areata in Taiwan: a case-control study. Br J Dermatol. 2012;166:525–31. 22049923 10.1111/j.1365-2133.2011.10714.x

[14] Alghamdi S Alsulami N Khoja S. Vitamin D supplementation ameliorates severity of major depressive disorder. J Mol Neurosci. 2020;70:230–5. 31836995 10.1007/s12031-019-01461-2

[15] Cui X Pertile R Liu P. Vitamin D regulates tyrosine hydroxylase expression: N-cadherin a possible mediator. Neuroscience. 2015;304:90–100. 26210580 10.1016/j.neuroscience.2015.07.048

[16] Acharya P Mathur MC. Oxidative stress in alopecia areata: a systematic review and meta-analysis. Int J Dermatol. 2020;59:434–40. 31875951 10.1111/ijd.14753

[17] Huang CM Lara-Corrales I Pope E. Effects of vitamin D levels and supplementation on atopic dermatitis: a systematic review. Pediatr Dermatol. 2018;35:754–60. 30284328 10.1111/pde.13639

[18] Ingram MA Jones MB Stonehouse W. Oral vitamin D3 supplementation for chronic plaque psoriasis: a randomized, double-blind, placebo-controlled trial. J Dermatolog Treat. 2018;29:648–57. 29480035 10.1080/09546634.2018.1444728

[19] Karagüzel G Sakarya NP Bahadir S. Vitamin D status and the effects of oral vitamin D treatment in children with vitiligo: a prospective study. Clin Nutr ESPEN. 2016;15:28–31. 28531780 10.1016/j.clnesp.2016.05.006

[20] Shamseer L Moher D Clarke M. Preferred reporting items for systematic review and meta-analysis protocols (PRISMA-P) 2015: elaboration and explanation [published correction appears in BMJ. 2016 Jul 21;354:i4086]. BMJ. 2015;350:g7647. 25555855 10.1136/bmj.g7647

[21] Olsen EA Hordinsky MK Price VH. Alopecia areata investigational assessment guidelines--part II. National alopecia areata foundation. J Am Acad Dermatol. 2004;51:440–7. 15337988 10.1016/j.jaad.2003.09.032

[22] Ross AC Taylor CL Yaktine AL., eds. Institute of Medicine (US) Committee to Review Dietary Reference Intakes for Vitamin D and Calcium. Dietary Reference Intakes for Calcium and Vitamin D. Washington (DC): National Academies Press (US) 2011. 21796828

[23] Olsen EA Roberts J Sperling L. Objective outcome measures: collecting meaningful data on alopecia areata. J Am Acad Dermatol. 2018;79:470–478.e3. 29128463 10.1016/j.jaad.2017.10.048 PMC7450487

[24] Jang YH Moon SY Lee WJ. Alopecia areata progression index, a scoring system for evaluating overall hair loss activity in alopecia areata patients with pigmented hair: a development and reliability assessment. Dermatology. 2016;232:143–9. 26757319 10.1159/000442816

[25] Liberati A Altman DG Tetzlaff J. The PRISMA statement for reporting systematic reviews and meta-analyses of studies that evaluate health care interventions: explanation and elaboration. PLoS Med. 2009;6:e1000100. 19621070 10.1371/journal.pmed.1000100 PMC2707010

[26] Higgins JP Altman DG Gøtzsche PC. The Cochrane Collaboration’s tool for assessing risk of bias in randomised trials. BMJ. 2011;343:d5928. 22008217 10.1136/bmj.d5928 PMC3196245

[27] Higgins JPT Green S, eds. Cochrane Handbook for Systematic Reviews of Interventions Version 5.1.0 [updated 2011]. The Cochrane Collaboration, 2011. Available at: www.cochrane-handbook.org.

[28] Mavridis D White IR. Dealing with missing outcome data in meta-analysis. Res Synth Methods. 2020;11:2–13. 30991455 10.1002/jrsm.1349 PMC7003862

[29] Higgins JP Thompson SG. Quantifying heterogeneity in a meta-analysis. Stat Med. 2002;21:1539–58. 12111919 10.1002/sim.1186

[30] Field AP Gillett R. How to do a meta-analysis. Br J Math Stat Psychol. 2010;63(Pt 3):665–94. 20497626 10.1348/000711010X502733

[31] Lin L Chu H. Quantifying publication bias in meta-analysis. Biometrics. 2018;74:785–94. 29141096 10.1111/biom.12817 PMC5953768

